# Transnasal endoscopic assisted dacryocystorhinostomy

**DOI:** 10.22336/rjo.2017.34

**Published:** 2017

**Authors:** Răzvan Hainăroșie, Irina Ioniță, Cătălina Pietroșanu, Silviu Pițuru, Mura Hainăroșie, Viorel Zainea

**Affiliations:** * ”Carol Davila” University of Medicine and Pharmacy, Bucharest, Romania; ** “Prof. Dr. Dorin Hociotă” Institute of Phonoaudiology and Functional ENT surgery, Bucharest, Romania

**Keywords:** endoscopic, transnasal, dacryocystorhinostomy

## Abstract

Transnasal endoscopic dacryocystorhinostomy is a good alternative for external DCR. It is considered a safe and efficient technique with successful results, comparable or even better than the external technique. Advanced knowledge of the endoscopic anatomy and the lacrimal system is necessary to perform the procedure safely, and to obtain good surgical outcomes.

The aim of the paper is to analyze the changes of the endoscopic technique and to improve it. The transnasal endoscopic dacryocystorhinostomy surgical technique is described in a “step by step” manner. Also the surgical technologies that can be used for this intervention are presented, focusing on the cold instruments.

## Introduction

In the last 20 years, an avalanche of developments, mainly in the endoscopic field, has taken place in ENT. Endoscopic sinus surgery developments were outstanding. Optical technologies and video chip advancements offered a perfect view of the endoscopic surgical field. Surgeons can perform more complex operations, safely and minimally invasive. 

Dacryocystorhinostomy (DCR) is the procedure that aims to create a direct drainage of the tears, opening the lacrimal sac directly into the nasal cavity in cases of nasolacrimal duct (NLD) blockage.

The procedure is over 100 years old, and it was described in the first century by Celsus. He created a new passage for the tears using a cautery at the level of the lacrimal bone. In the second century, Galen described the same procedure.

In the eighteenth century, Woolhouse imagined a technique to open the lacrimal sac into the maxillary sinus. That method is somehow similar to the modern DCR. He removed the lacrimal sac and placed a stent of gold or silver. Some of the authors reported success in almost 70-85% of the cases.

The first modern description of an external DCR was published in 1904, while the modified version of Dupuy-Dutemps and Bourguet remain the gold standard for the treatment of NLD obstruction [**1**].

In 1893, Caldwell described for the first time the endonasal approach of an endoscopic DCR [**2**]. In 1989, Meiring and Mc Donogh described the modern DCR. In our days, transnasal endoscopic DCR is the primary treatment for lacrimal obstruction or revision surgery before external DCR.

Indications of transnasal endoscopic DCR include patients with NLD demonstrated obstruction in cases of facial trauma, failed external DCR, adolescents with anatomical variations or congenital disorders of the lacrimal pathway. The scar or adhesions can be removed in cases of previous failure of the endoscopic DCR.

Relative contraindications of endoscopic DCR are acute dacryocystitis and anticoagulant treatment that cannot be stopped.

Absolute contraindications are tumors of the lacrimal sac.

The aim of the paper was to analyze the modifications of the endoscopic technique and to improve it.

## Material and method

The transnasal endoscopic dacryocystorhinostomy was performed under general anesthesia with transoral intubation. 

Cotton pads moisturized in adrenalin solution were placed at the level of the medial meatus and inferior turbinate, under endoscopic control.

Septoplasty is performed if a septal deviation blocks the access to the medial meatus.

The middle meatus was localized by using a 0-degree rigid endoscope. Moreover, the middle turbinate was gently medialized.

The following endoscopic landmarks have to be identified: middle turbinate, lacrimal crest, uncinate process, bulla ethmoidalis.

Some authors recommend the head of the middle turbinate as a landmark, but, in our opinion, it is not a safe landmark due to the high variability of this structure.

The uncinate process was located to identify the lacrimal sac region and the anterior of the lacrimal crest was defined.

Many techniques were described by using multiple mucoperichondrial flaps from the nasal mucosa. After multiple tryouts, only one flap is currently used. 

**Fig. 1:**
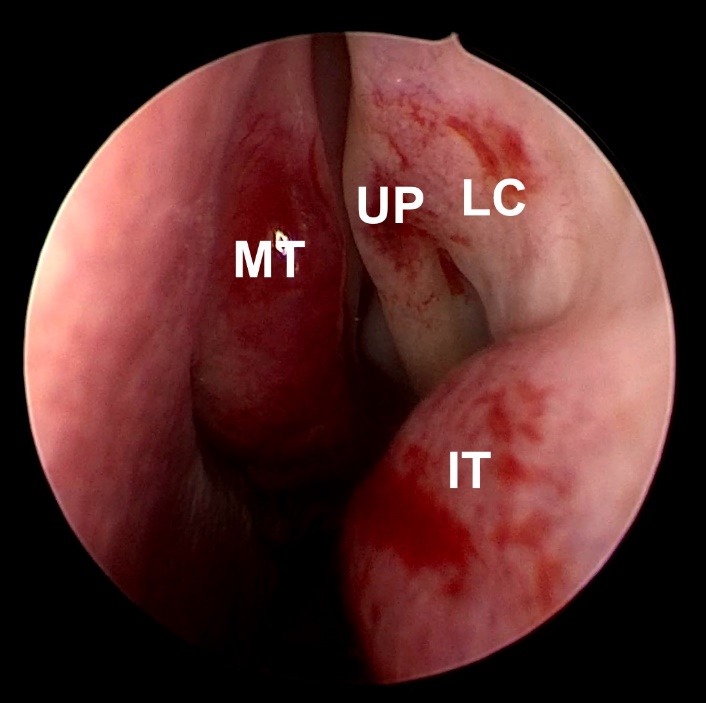
Endoscopic landmarks; MT- middle turbinate; UP - uncinate process; LC -lacrimal crest; IT - inferior turbinate

A vertical incision was performed at the level of the anterior limit of the lacrimal crest, starting at the level of the middle turbinate insertion and downwards to the superior part of the inferior turbinate.

**Fig. 2:**
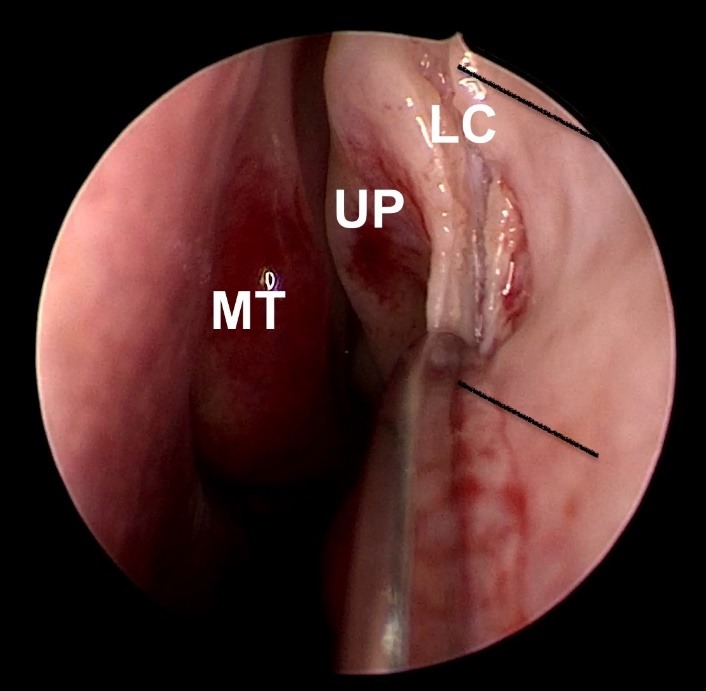
Incision on the lacrimal crest and anterior flap design; MT - middle turbinate; UP - uncinate process; LC - lacrimal crest

Two parallel horizontal incisions were made at the extremities of the vertical incisions from the anterior up to the posterior part of the lacrimal crest.

The mucoperiosteal flap was elevated anteriorly and the lacrimal bone was exposed.

The bone removed was represented by the maxillary branch and lacrimal bone. In cases of agger nasi rich pneumatization, some anterior ethmoidal cells can be removed as well.

**Fig. 3:**
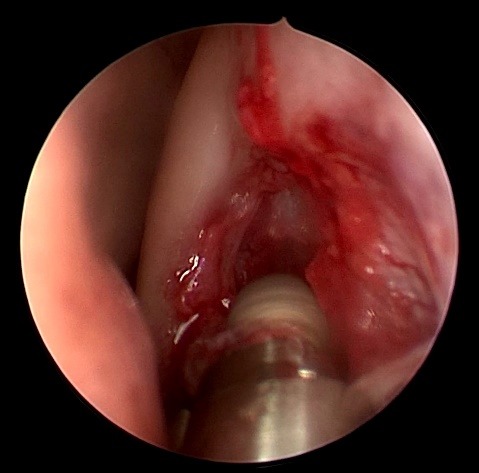
Drilling the lacrimal bone

A high-speed drill was used to remove the bone. Some authors recommend the use of piezotome, a device that removes only bone and does not harm the soft tissue, with minimal thermal effect.

We removed as much bone as we could vertically.

A Bowman cannula was inserted into the inferior canaliculus to identify the medial wall of the lacrimal sac. 

**Fig. 4:**
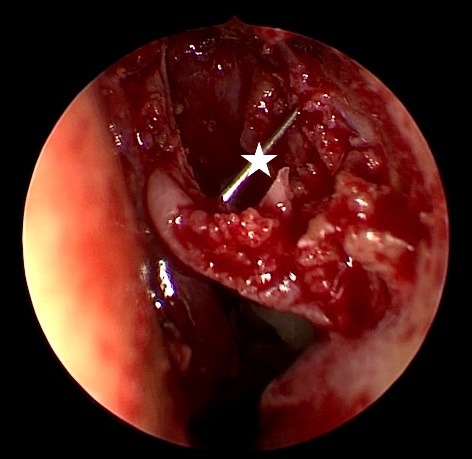
Bowman cannula insertion will better identify the projection of the lacrimal sac. An incision can be performed safely

The medial wall of the lacrimal sac was incised with the endoscopic knife in an “H” shape.

The two vertical flaps from the lacrimal sac were exposed until the posterior flap came in close contact with the nasal mucosa located at the posterior limit of the lacrimal crest.

**Fig. 5:**
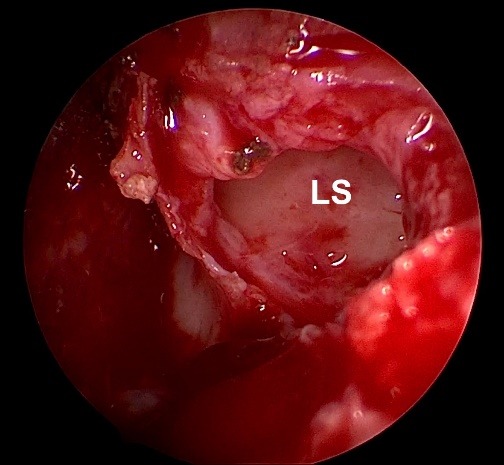
Wide opening of the lacrimal sac, no stenting required

The nasal mucosal anterior flap was shortened with the shaver until it reached the anterior flap from the lacrimal sac. 

A piece of Gelaspone was placed to stabilize the flaps. 

No stenting was performed.

No nasal packing was necessary.

Postoperative care was done by using saline solution and oil drops to clean the nose.

The first visit was at 14 days, then at one month and two months.

## Results and discussions

Our success rate in 37 patients was 91.89% after the first surgery and 97.3% after the second surgery. No external DCR was performed.

First, it was believed that the external DCR would obtain better results than the endoscopic DCR, but recent studies showed that the results are comparable, maybe better in endoscopic DCR [**3**].

The advantages in endoscopic DCR include the fact that it does not disturb the lacrimal pump system [**4**]. Also, all surrounding structures were visualized.

Better esthetic results are obtained without a skin incision.

Less operative bleeding was reported together with no scar to the external canthus area and orbicularis muscle.

A disadvantage of the endoscopic DCRS is that a lacrimal neoplasia cannot be excluded; if a suspicion is raised, a biopsy must be performed [**5**].

LASER or radiofrequency assisted DCR was described, but we do not recommend that technique because of the thermal damage produced to the tissue and the additional cost of the procedure. We recommend using “cold” instruments.

Taking into account that endoscopic and external DCR have comparable results, from an ethical point of view, the surgeon has to present both options (with advantages, disadvantages, risks, benefits, possible complications) to the patient. The esthetic result of the procedure must be addressed, and the patient can choose the most convenient procedure. The informed consent is obtained before surgery. 

Also, the surgeon has to be aware of the limits imposed by his/ her medical specialty, and always have in mind an improvement of the patients’ health as a result of the treatment.

## Conclusion

Transnasal endoscopic dacryocystorhinostomy is a safe and efficient technique with a success result comparable or even better than external DCR. 

Advanced knowledge of endoscopic anatomy and lacrimal system is necessary to perform the procedure safely. In case of failure, the procedures can be repeated, or external DCR can be conducted. 

In our opinion, “cold” instruments are the best choice to perform the surgery. 

Further studies must be made concerning the use of piezoelectric surgery in lacrimal bone removal. That technology offers a “cold” removal of the bone without damaging the soft tissue, reducing scar formation, but the operation will take longer since the speed is low with that type of technology.

**Acknowledgement**

All the authors have contributed equally to this paper.

## References

[1] Dubey SP, Munjal VR (2014). Endoscopic DCR: How To Improve The Results. Indian Journal of Otolaryngology and Head & Neck Surgery.

[2] Muscatello L, Giudice M, Spriano G, Tondini L (2005). Endoscopic dacryocystorhinostomy: personal experience. Acta Otorhinolaryngologica Italica.

[3] Tsirbas A, Wormald PJ (2003). Endonasal dacryocystorhinostomy with mucosal flaps. Am J Ophthalmol.

[4] Marcet MM, Kuk AK, Phelps PO (2014). Evidence-based review of surgical practices in endoscopic endonasal dacryocystorhinostomy for primary acquired nasolacrimal duct obstruction and other new indications. Curr Opin Ophthalmol.

[5] Jawaheer L, MacEwen CJ, Anijeet D (2017). Endonasal versus external dacryocystorhinostomy for nasolacrimal duct obstruction. Cochrane Database Syst Rev.

